# The Prevalence, Severity, and Impact of Post-COVID Persistent Fatigue, Post-Exertional Malaise, and Chronic Fatigue Syndrome

**DOI:** 10.1007/s11606-022-07882-x

**Published:** 2022-11-10

**Authors:** Mayssam Nehme, Francois Chappuis, Laurent Kaiser, Frederic Assal, Idris Guessous

**Affiliations:** 1 Division of Primary Care Medicine, Geneva University Hospitals, Geneva, Switzerland; 2 Faculty of Medicine, University of Geneva, Geneva, Switzerland; 3 Division of Tropical and Humanitarian Medicine, Geneva University Hospitals, Geneva, Switzerland; 4 Division of Infectious diseases, Geneva University Hospitals, Geneva, Switzerland; 5 Geneva Center for Emerging Viral Diseases, Geneva University Hospitals, Geneva, Switzerland; 6 Division of Laboratory Medicine, Laboratory of Virology, Geneva University Hospitals, Geneva, Switzerland; 7 Division of Neurology, Geneva University Hospitals, Geneva, Switzerland

## BACKGROUND

Fatigue is common after viral infections, including SARS-CoV-2.^1^ Our purpose was to report the prevalence and impact of persistent fatigue 6 months after SARS-CoV-2 infection, considering post-exertional malaise^2^ and criteria for chronic fatigue syndrome.^3^

## METHODS

Since March 2020, individuals tested for SARS-CoV-2 at the Geneva University Hospitals outpatient testing center benefit from remote ambulatory follow-up (COVICARE).^1^ This study included all individuals tested between March 2020 and December 2020 and whose follow-up was at 6 months or more after their test date.

Follow-up included questions about the prevalence of symptoms (yes/no) and their severity using a Likert scale (mild, moderate, or severe). Fatigue was assessed using the Eastern Cooperative Oncology Group (ECOG) scale and the Chalder fatigue scale.^4^ The Chalder fatigue scale was scored using the 4-item Likert and the bimodal scoring schemes. A score of ≥ 4 on bimodal scoring indicated severe fatigue. The DePaul brief questionnaire^5^ was used to identify post-exertional malaise and criteria for chronic fatigue syndrome. The Sheehan Disability Scale was used to assess functional impairment. Reduced work capacity was defined as missing days off work or having a reduced productivity on the Sheehan disability scale. Comorbidities were considered present if pre-existing prior to SARS-CoV-2 infection. Statistical analysis included descriptive comparisons of percentages using chi-square tests and Student’s *t* test.

## RESULTS

Overall, 5515 individuals participated in this study (response rate 70.7%), with 5406 participants at 6 months or more after their test date. A total of 1497 (27.7%) participants had a documented positive SARS-CoV-2 test and were ultimately included in the study. The median time for follow-up was 225 days (interquartile range 207–398). Respectively, fatigue was reported by 17.2%, post-exertional malaise by 8.2%, and the presence of criteria for chronic fatigue syndrome by 1.1% of SARS-CoV-2-positive individuals, compared to 8.9%, 3.5%, and 0.5% of SARS-CoV-2-negative individuals. Characteristics are presented in Table 1.

**Table 1:** Baseline Characteristics of SARS-CoV-2-Positive Participants at 6 Months or More After Their Infection (*n* = 1497)

	No fatigue (*n* = 1239)	Fatigue (*n* = 258)	Total (*n* = 1497)	*P* value
	*n* (%)	*n* (%)	*n* (%)	
Age categories				0.271
Below 40	505 (40.8)	102 (39.5)	607 (40.5)	
40–59	565 (45.6)	129 (50.0)	694 (46.4)	
60 and above	169 (13.6)	27 (10.5)	196 (13.1)	
Sex				< 0.001
Male	566 (45.6)	82 (31.8)	645 (43.2)	
Female	673 (54.4)	176 (68.2)	847 (56.8)	
Education				0.124
Primary	47 (4.7)	10 (4.9)	57 (4.8)	
Apprenticeship	108 (10.9)	20 (9.9)	128 (10.7)	
Secondary	125 (12.6)	35 (17.2)	160 (13.4)	
Tertiary	643 (64.9)	117 (57.6)	760 (63.7)	
Other	55 (5.6)	15 (7.4)	70 (5.9)	
Prefer not to answer	12 (1.2)	6 (3.0)	18 (1.5)	
Profession				0.588
None	88 (8.9)	15 (7.4)	103 (8.6)	
Unskilled workers	39 (3.9)	5 (2.5)	44 (3.7)	
Skilled workers	153 (15.5)	40 (19.7)	193 (16.2)	
High-grade skilled workers	255 (25.8)	58 (28.6)	313 (26.2)	
Professional managers	284 (28.7)	52 (25.6)	336 (28.2)	
Other	159 (16.1)	30 (14.8)	189 (15.8)	
Prefer not to answer	12 (1.2)	3 (1.5)	15 (1.3)	
Civil status				0.151
Single	200 (17.9)	47 (18.2)	247 (18)	
In couple, not married	258 (23.1)	73 (28.3)	331 (24.1)	
Married or registered partnership	546 (48.9)	107 (41.5)	653 (47.5)	
Divorced or separated	98 (8.8)	28 (10.9)	126 (9.2)	
Widowed	11 (1.0)	1 (0.4)	12 (0.9)	
Other	3 (0.3)	2 (0.8)	5 (0.4)	
Have children	704 (63.1)	157 (60.9)	861 (62.7)	0.505
Living status				0.023
Alone	195 (17.5)	52 (20.2)	247 (18.0)	
Single parent with children	55 (4.9)	21 (8.1)	76 (5.5)	
In couple, without children	258 (23.1)	56 (21.7)	314 (22.9)	
In couple, with children	503 (45.1)	95 (36.8)	598 (43.6)	
Cohabitation with other people	104 (9.3)	34 (13.2)	138 (10.1)	
Work situation				0.002
Salaried	815 (73.2)	186 (72.1)	1,001 (73)	
Retired	86 (7.7)	11 (4.3)	97 (7.1)	
Student or training	80 (7.2)	19 (7.4)	99 (7.2)	
Independent worker	52 (4.7)	11 (4.3)	63 (4.6)	
Homemaker	23 (2.1)	5 (1.9)	28 (2)	
Unemployed	25 (2.2)	15 (5.8)	40 (2.9)	
Disability	7 (0.6)	7 (2.7)	14 (1.0)	
Other	25 (2.2)	4 (1.6)	29 (2.1)	
Contract situation				< 0.001
Short-term contract	85 (9.2)	26 (12.4)	111 (9.8)	
Long-term contract	655 (71.2)	167 (79.9)	822 (72.8)	
Subsidized contract	1 (0.1)	1 (0.5)	2 (0.2)	
Training	19 (2.1)	2 (1.0)	21 (1.9)	
Not concerned	40 (4.3)	9 (4.3)	49 (4.3)	
Other	120 (13.0)	4 (1.9)	124 (11.0)	
Work activity				0.047
Not working	21 (2.2)	4 (1.8)	25 (2.1)	
Less than 30%	22 (2.3)	4 (1.8)	26 (2.2)	
30–49%	33 (3.4)	7 (3.2)	40 (3.4)	
50–79%	103 (10.6)	38 (17.3)	141 (11.9)	
80–99%	172 (17.8)	48 (21.8)	220 (18.5)	
100%	608 (62.7)	115 (52.3)	723 (60.8)	
Prefer not to answer	10 (1.0)	4 (1.8)	14 (1.2)	
Smoking status				0.142
Non-smoker	715 (59.6)	145 (56.2)	860 (59.0)	
Ex-smoker	315 (26.3)	68 (26.4)	383 (26.3)	
Current smoker	141 (11.8)	36 (14.0)	177 (12.1)	
Prefer not to answer	28 (2.3)	9 (3.5)	37(2.5)	
Activity level				< 0.001
None	143 (11.9)	58 (22.5)	201 (13.8)	
Partial	605 (50.5)	145 (56.2)	750 (51.5)	
Full physical activity	438 (36.5)	51 (19.8)	489 (33.6)	
Prefer not to answer	13 (1.1)	4 (1.6)	17 (1.2)	
Vaccination status				0.001
No vaccination	171 (14.6)	19 (7.4)	190 (13.3)	
Partially vaccinated (1 dose)	212 (18.1)	60 (23.3)	272 (19.0)	
Fully vaccinated (at least 2 doses)	782 (63.1)	177 (68.6)	959 (64.1)	
Prefer not to answer	8 (0.7)	2 (0.8)	10 (0.7)	
Hospitalization	62 (5.3)	22 (8.8)	84 (5.9)	0.086
Reinfection	120 (9.7)	29 (11.2)	149 (10.0)	0.448
BMI (kg/m^2^)				0.063
Below 18.5	33 (3.0)	7 (2.9)	40 (3.0)	
18.5–24.9	579 (53.5)	132 (53.9)	711 (53.6)	
25–29.9	265 (24.5)	74 (30.2)	339 (25.5)	
30–34.9	183 (16.9)	25 (10.2)	208 (15.7)	
35 and above	22 (2.0)	7 (2.9)	29 (2.2)	
Symptoms at testing				0.002
Pauci-symptomatic	219 (23.7)	26 (13.5)	245 (21.9)	
Have several symptoms	707 (76.3)	167 (86.5)	874 (78.1)	
Comorbidities				
None	665 (53.7)	115 (44.6)	780 (52.1)	0.012
Obesity or overweight	159 (12.8)	32 (12.4)	191 (12.8)	0.504
Hypertension	81 (6.5)	16 (6.2)	97 (6.5)	0.168
Diabetes	18 (1.5)	7 (2.7)	25 (1.7)	0.164
Respiratory disease	31 (2.5)	9 (3.5)	40 (2.7)	0.455
Cardiovascular disease	24 (1.9)	6 (2.3)	30 (2.0)	0.540
Headache disorders	107 (8.6)	30 (11.6)	137 (9.2)	< 0.001
Chronic pain or fibromyalgia	5 (0.4)	4 (1.6)	9 (0.6)	0.028
Hyperthyroidism	5 (0.4)	3 (1.2)	8 (0.5)	0.054
Hypothyroidism	22 (1.8)	8 (3.1)	30 (2.0)	0.029
Anemia	18 (1.5)	8 (3.1)	26 (1.7)	0.020
Chronic fatigue	13 (1.0)	6 (2.3)	19 (1.3)	0.046
Cognitive disorders	25 (2.0)	3 (1.2)	28 (1.9)	0.395
Sleep disorders	78 (6.3)	20 (7.8)	98 (6.5)	0.361
Depression	29 (2.3)	9 (3.5)	38 (2.5)	0.542
Anxiety	41 (3.3)	12 (4.7)	53 (3.5)	0.990
Irritable bowel syndrome	40 (3.2)	9 (3.5)	49 (3.3)	0.309
Rheumatologic disorders	49 (4.0)	6 (2.3)	55 (3.7)	0.727
Tendinitis	25 (2.0)	8 (3.1)	33 (2.2)	0.022

Out of SARS-CoV-2-positive participants with fatigue (*n* = 258), 35.3% had moderate to severe limitations on the ECOG scale, and 83.0% had a score ≥ 4 on the Chalder fatigue scale. The Chalder fatigue scale revealed a mean score of 19 out of 33, SD 5.4, and a mean score of 6.7 out of 11, SD 3.3 using bimodal scoring. After adjusting for age and sex, 47.7% of SARS-CoV-2-positive individuals with fatigue at 6 months or more had the frequency and severity criteria for post-exertional malaise, and 6.2% had criteria for chronic fatigue syndrome.

**Figure 1:**
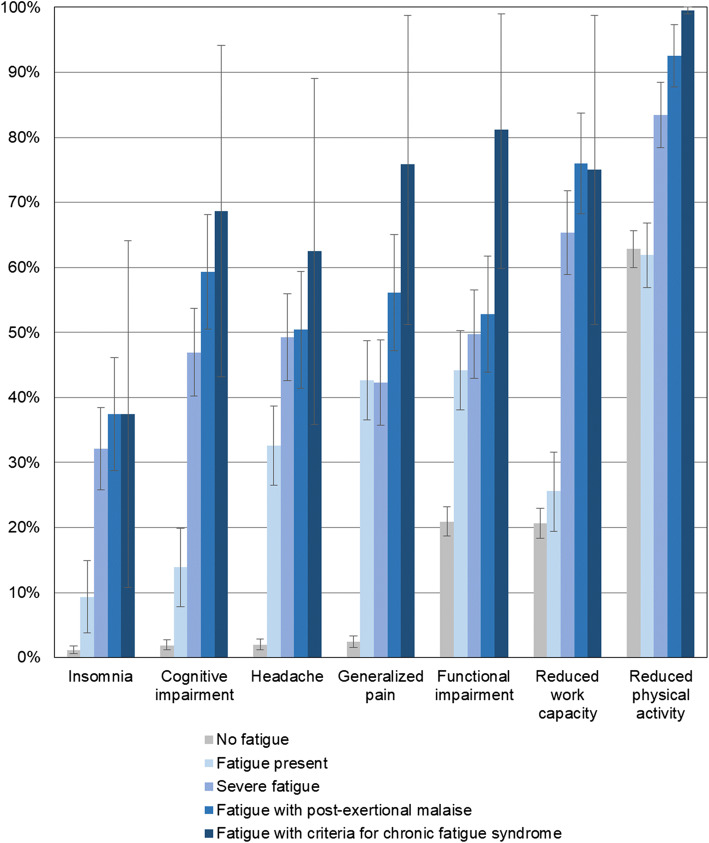
The prevalence of newly developed insomnia, cognitive impairment, headache, generalized pain, and functional and physical impairment stratified by fatigue severity including post-exertional malaise and criteria for chronic fatigue syndrome in SARS-CoV-2-positive individuals at 6 months or more after their infection (*n* = 1497)*. Prevalence is adjusted for age and sex. Only newly reported symptoms and sequelae after SARS-CoV-2 infection were included in this analysis. Severe fatigue is defined as a Chalder fatigue scale score ≥ 4. The DePaul brief questionnaire evaluated the frequency and severity of symptoms characterizing post-exertional malaise including heaviness or drowsiness after exercise, pain, fatigue, and exhaustion after minimal effort, as well as the time required for recovery. Using a Likert scale, a score of 2 or more on the frequency (5 questions) and severity (5 questions) of symptoms indicated post-exertional malaise. If recovery required more than 14 h after minimal physical or mental activity, the questionnaire was positive for chronic fatigue syndrome.

Individuals had a higher prevalence of insomnia, cognitive impairment, headaches, generalized pain, functional impairment, reduced work capacity, and decreased physical activity, after SARS-CoV-2 infection. The prevalence of these sequelae was adjusted for age and sex and was increasingly higher with severe fatigue, with post-exertional malaise, or when criteria for chronic fatigue syndrome were present (Fig. 1).

## DISCUSSION

Fatigue is the most common and persistent post-COVID symptom. The spectrum of fatigue severity in post-COVID individuals ranges from feeling tired to having severe fatigue, post-exertional malaise, or criteria for chronic fatigue syndrome with an increasing impact on health, functional capacity, and physical activity.

Almost half of individuals experiencing fatigue at 6 months after the infection had post-exertional malaise, and 6.2% had criteria for chronic fatigue syndrome, prompting physicians to consider pacing as a management option, in the absence of other treatment options at this stage. SARS-CoV-2 infection was positively associated with fatigue and post-exertional malaise. Results showed that individuals with fatigue were more likely to be vaccinated. This was partially explained by the baseline distribution as older individuals and those with more comorbidities were more likely to get vaccinated.

Results compare to recent reviews showing an overlap between post-COVID condition and chronic fatigue syndrome.^6^ Our study graded post-COVID fatigue by severity in correlation with functional capacity, and showed the high prevalence of post-exertional malaise.

Limitations include the self-reported nature of this follow-up with individuals infected in 2020 and follow-up in 2021, lacking comparisons to individuals infected with other variants. Additionally, this study considered having received at least 2 doses as full vaccination, a concept that continues to evolve with time.

Physicians, employers, and insurance companies should address fatigue on a spectrum, accounting for the correlated functional impairment, decreased activity levels, and potentially poorer quality of life.

## Declarations

### Statement

We confirm that the manuscript has been read and approved by all named authors and that there are no other persons who satisfied the criteria for authorship but are not listed. We further confirm that this work has been conducted with the ethical approval of the Cantonal Research Ethics Commission of Geneva, Switzerland, and that the approvals are acknowledged within the manuscript.

## References

[1] **Nehme M, Braillard O, Chappuis F, Courvoisier DS, Guessous I.** Prevalence of symptoms more than seven months after diagnosis of symptomatic COVID-19 in an outpatient setting. Ann Intern Med. 2021. 10.7326/M21-0878. 10.7326/M21-0878 PMC8280535 34224254

[2] Centers for Disease Control and Prevention, National Center for Emerging and Zoonotic Infectious Diseases (NCEZID), Division of High-Consequence Pathogens and Pathology (DHCPP). https://www.cdc.gov/me-cfs/healthcare-providers/clinical-care-patients-mecfs/treating-most-disruptive-symptoms.html. Access 20 Mar 2022.

[3] Institute of Medicine of the National Academies. Beyond myalgic encephalomyelitis/chronic fatigue syndrome: redefining an illness. Report Brief, February 2015. 25695122

[4] **Chalder T, Berelowitz G, Pawlikowska T, Watts L, Wessely S, Wright D, Wallace EP.** Development of a fatigue scale. J Psychosom Res. 1993;37(2):147-53. 10.1016/0022-3999(93)90081-p. 10.1016/0022-3999(93)90081-p 8463991

[5] **Cotler J, Holtzman C, Dudun C, Jason LA.** A brief questionnaire to assess post-exertional malaise. Diagnostics (Basel). 2018;8(3):66. 10.3390/diagnostics8030066. 10.3390/diagnostics8030066 PMC6165517 30208578

[6] **Wong TL, Weitzer DJ.** Long COVID and myalgic encephalomyelitis/chronic fatigue syndrome (ME/CFS)-a systemic review and comparison of clinical presentation and symptomatology. Medicina (Kaunas). 2021;57(5):418. Published 2021 Apr 26. 10.3390/medicina57050418. 10.3390/medicina57050418 PMC8145228 33925784

